# The physiological consequences of breath-hold diving in marine mammals: the Scholander legacy

**DOI:** 10.3389/fphys.2012.00473

**Published:** 2012-12-19

**Authors:** Andreas Fahlman

**Affiliations:** Department of Life Sciences, Texas A&M University-Corpus ChristiCorpus Christi, TX, USA

Most of the physiological traits used by marine mammals to perform long and deep breath-hold dives were described in Scholander's seminal paper in 1940. Since then, several studies have provided an improved understanding of the mechanistic basis of the mammalian diving response (Scholander, 1940, 1963; Mottishaw et al., 1999; Fahlman et al., 2011), the aerobic dive limit (ADL) (Kooyman et al., 1980; Butler and Jones, 1997; Davis and Kanatous, 1999; Horning, 2012), and management of respiratory gases (Boutilier et al., 2001; Fahlman et al., 2008a; Hooker et al., 2009; Kvadsheim et al., 2012), but many questions remain. Some widely-accepted ideas actually lack experimental confirmation, and a variety of marine mammal species, potentially novel models for elucidating new diving adaptations, have not been adequately studied. The aim of this Frontiers Special Topic is to provide a synthesis of the current knowledge of the physiological responses that may explain the varied diving behavior of marine mammals. We strove to include contributions that challenge current ideas, and which propose new hypotheses, utilize new experimental approaches, and explore new model species.

Much work has been dedicated to understanding the ADL and how a species can manage its foraging within its ADL. The ADL was originally defined as the length of time an animal could remain submerged before the post-dive blood lactate levels began to increase (Kooyman et al., 1980). The calculated aerobic dive limit (cADL) was later conceived to estimate the maximum duration of aerobic metabolism by dividing the total usable O_2_ stores by the rate of O_2_ consumption (metabolic rate, Butler and Jones, 1997). While most species appear to dive well within their cADL, others appear to exceed the cADL on a regular basis (Costa et al., 2001). Horning proposes an interesting method to investigate the plasticity of the functional ADL using constraint lines, which may help improve our understanding of the link between behavior and physiology (Horning, 2012). On a physiological level, it is possible that dives that appear to be beyond the cADL are actually attributable to underestimating the usable O_2_ stores, or overestimating the metabolic costs of diving and foraging (Hurley and Costa, 2001; Fahlman et al., 2008b; Ponganis et al., 2011). A study suggests that elephant seals possess extreme hypoxia tolerance and make use of their entire blood O_2_ store during diving (Meir et al., 2009; Ponganis et al., 2011). The use of the spleen to increase hematocrit during diving has been shown to enhance breath-hold capacity in humans (Schagatay et al., 2012) and in marine mammals (Cabanac, 2000; Thornton et al., 2001). It may be that previous analyses of cADL have missed these sources of usable O_2_ (Meir and Ponganis, 2009; Ponganis et al., 2011).

Logistical constraints have made it difficult to estimate metabolic rate in foraging animals (Ponganis et al., 2011). Variation in prey density or other environmental factors may alter metabolic costs of foraging. It has been hypothesized that alteration in prey species may affect the nutritional status of the predator (Rosen, 2009). Trumble and Kanatous (2012) argue that the metabolic stoichiometry between O_2_ and ATP is affected by the lipid composition of the diet. As the lipid composition varies between prey species and seasons, the ingested food may alter the foraging efficiency through changes in the metabolic burden while underwater.

Weingartner et al. (2012) have shown that increased thyroid hormone levels elevate the metabolic rate during diving in harbor seals and result in higher post-dive lactate levels. This suggests that thyroid hormone could be important in modulating metabolic rate to fit the dive conditions. The higher metabolic rate resulted in a more pronounced reduction in heart rate during the dive. This provides an interesting link between endocrine and neural control of the physiological responses during diving. The hyperthyroid animals, with a more extreme diving bradycardia, may be indirect evidence of the O_2_ conserving effect of the diving response (Weingartner et al., 2012). The diving response is believed to be a conserved physiological trait, which includes diving-induced bradycardia, peripheral vasoconstriction, and altered blood flow distribution (Mottishaw et al., 1999; Fahlman et al., 2011). While our understanding of the central control of the diving response is limited (McCulloch, 2012; Panneton et al., 2012), the bradycardia results in reduced cardiac work. It is not clear whether the reduced work is sufficient to significantly lower the overall metabolic burden, or whether the response serves other purposes. An alternate hypothesis is that the primary role of the diving bradycardia is to regulate the degree of hypoxia in skeletal muscle so that blood and muscle O_2_ stores can be used more efficiently (Davis and Kanatous, 1999).

If marine mammals generally dive within their cADL, what other physiological constraints may limit diving? Scholander suggested that alveolar collapse (commonly called lung collapse) would limit uptake of N_2_ and reduce the likelihood of decompression sickness (DCS, Scholander, 1940). However, necropsy reports from mass stranded whales indicated DCS-like symptoms (Jepson et al., 2003; Fernández et al., 2005). A more recent study has shown that the gas bubble composition in stranded whales is similar to that from land mammals suffering DCS in experimental dive models (Bernaldo De Quirós et al., 2012). Imaging work in both live and stranded marine mammals indicates that they live with elevated inert gas tensions that cause bubbles to form under certain circumstances (Dennison et al., 2012). This raises some interesting questions: are marine mammals ever at risk of DCS, and if so, could N_2_ accumulation limit dive performance (Hooker et al., 2009; Kvadsheim et al., 2012; Sivle et al., 2012)? The estimated end-dive N_2_ levels suggest that a significant proportion of marine mammals should experience DCS symptoms if their responses to elevated N_2_ are physiologically similar to those of humans and various species of land mammals used in diving simulations (Hooker et al., 2009). Our understanding of the anatomy and physiology of marine mammals is not well-defined in this regard. The DCS model assumptions are based on data from widely different species, which may explain the elevated predictions for marine mammals. A recent study by Costidis and Rommel (2012) provides data on the vascular anatomy in bottlenose dolphins, suggesting that certain adipose tissue compartments may be highly vascularized. The ability to exchange gases in these compartments would vastly alter our understanding of how these species manage gases underwater, and provide interesting research challenges for the future.

Since the initial studies by Scholander in the 1940's, physiologists have been fascinated by the diving traits of marine mammals, and there is a large heritage not only from Scholander, but also from other classical work following this pioneer. While most of the physiological and biochemical traits were suggested Scholander and Irving, few have received as much study as the diving response and O_2_ management. The contributions to this special topic have shown that the field of diving physiology has recently entered a phase of renewed discovery that is revealing more secrets of the natural responses observed in marine mammals. While there is still a lot more to learn this special topic has focused on work progressing from this heritage, instead of re-inventing knowledge. What is becoming clear is that marine mammals may be a useful model system to understand physiological challenges in extreme environments.
